# 

**Published:** 1847-08

**Authors:** 


*To Correspondents.*—Our correspondent who has sent us an article on "Medical authorship," is respectfully informed that we reluctantly decline its publication, from its want of application to the subjects which, for the most part, our readers require at our hands. We have received various intimations that, for our work to be acceptable, its contents must have direct or indirect relations to practical medicine. *Cui bono* is the motto of the day; and perhaps in no province of science or art more than in medicine is utilitarianism more essential as respects the estimation of Practitioners generally. The author of the article referred to will, we trust, appreciate our motives. We are by no means insensible to the ability displayed in its composition, and predict for him high reputation as a writer when time and experience have chastened somewhat the enthusiasm of youth. We hope when he enters the arena of *Medical Authorship* he will avoid the faults which he so justly ponrtrays.

We must decline the report of a case of malignant disease of tongue and mouth, although it is not without interest, and is drawn up with more than ordinary ability. Our reason for declining it is, that it conveys reflections upon the professional skill of the medical gentlemen who had preceded in the treatment of the case. We presume this occurred through inadvertence, but our correspondent must agree with us that it would not less be a violation of medical ethics in a published communication, than in an oral expression of opinion.

The letter signed *Sophronistes* we presume was not intended for publication, but was designed for our especial benefit. The burlesque is well done, and perhaps the satire is merited; but the writer has doutless too much kindness of heart to wish to see it in print.

We take this occasion to say, generally, with regard to contributions to our pages, that not the least important of the objects for which our journal was instituted, is to afford a medium for the free interchange of views, and the communication of facts for the benefit of science and the profession. We would not only invite, but earnestly solicit contributions, and, provided our correspondents confine themselves to topics of practical interest and importance, avoiding personalities, we are not disposed to be over fastidious in accepting their productions. In exercising our privilege of declining articles which are objectionable in some of their features, or which do not seem adapted to the wishes of our readers, albeit creditable in a literary point of view, we trust our correspondents will neither be dissatisfied, nor deterred from continuing their efforts. We wish we could induce a greater number of our friends to become writers, both for their own satisfaction and our advantage—but on this subject we will have something farther to say hereafter.

---

*Medical Meeting.*—At the Semi-Annual meeting of the Genesee County Medical Society, held at the American Hotel in the village of Batavia, on the 2d Tuesday of June, 1847, it having come to the knowledge of this Society, that Drs. Almon Pratt and Alfred Wilcox, of Le Roy, have connected themselves with Quackery in its worst form, it was therefore, on motion of Dr. Ford, unanimously

*Resolved,* That the two above named individuals be and are hereby expelled from this society.

*Resolved,* That the above resolution be published in the papers of this county, under the direction of the Secretary.

H. GANSON, Sec'y.

---

Notice of the work by Prof. G. B. Wood on the Practice of Medicine, is crowded out of this number, and will appear in our next.

